# Spray-induced gene silencing for crop protection: recent advances and emerging trends

**DOI:** 10.3389/fpls.2025.1527944

**Published:** 2025-02-20

**Authors:** Can Chen, Muhammad Imran, Xianyang Feng, Xihui Shen, Zhongke Sun

**Affiliations:** ^1^ Key Laboratory of Plant Genetics and Molecular Breeding, Henan Key Laboratory of Crop Molecular Breeding & Bioreactor, College of Life Science and Agronomy, Zhoukou Normal University, Zhoukou, China; ^2^ School of Biological Engineering, Henan University of Technology, Zhengzhou, China; ^3^ State Key Laboratory for Crop Stress Resistance and High-Efficiency Production, Shaanxi Key Laboratory of Agricultural and Environmental Microbiology, College of Life Sciences, Northwest A&F University, Yangling, China

**Keywords:** biopesticides, SIGS, dsRNA, crop protection, nanotechnology, RNAi, sustainable agriculture

## Abstract

The RNA-based spray-induced gene silencing (SIGS) technology represents an ecologically sustainable approach to crop protection and pathogen management. Following the recent approval of Ledprona as the first sprayable double-stranded RNA (dsRNA) biopesticide by the EPA at the end of 2023, SIGS has emerged as a focal point in both academic and industrial sectors. This review analyzes recent advances and emerging trends in SIGS. The application of SIGS for crop protection, including the control of insects, fungal pathogens, and viruses, is briefly summarized. Distinguishing this review from others, we delve into practical aspects of the technology, such as the selection and screening of target genes, large-scale production methods, and delivery systems, highlighting major advancements in these areas and also addressing the remaining questions and issues, particularly concerning safety concerns and controlling harmful weeds. Finally, this review emphasizes the emerging trends in SIGS technology, particularly its integration with nanotechnology and other methodologies. Collectively, the rapid progress in SIGS studies is poised to accelerate the maturation and application of this technology.

## 1 Introduction

It was demonstrated that some pathogens can deliver small RNAs (sRNAs) into host cells to suppress host immunity. Conversely, hosts also transfer sRNAs into pathogens and pests to inhibit their virulence (Wang et al., 2016, 2017). Travel of sRNAs between interacting organisms can induce gene silencing in the counter party, a mechanism termed cross -kingdom RNA interference (RNAi) or trans-kingdom RNAi (Cai et al., 2018a). As an example, trans-kingdom RNAi has been recently proposed for potential management of *Fusarium* wilt disease (Huang et al., 2025). In fact, trans-kingdom RNAi can be achieved either by host-induced gene silencing (HIGS) or spray-induced gene silencing (SIGS). The HIGS technology has become an important disease-control method by transgenic expression of pathogen gene-targeting double-stranded RNA (dsRNA) in plants (Cai et al., 2018b; Koch and Wassenegger, 2021). However, transgenic approaches have limitations during development and promotion. To circumvent the release of genetic modified plants, spray-induced gene silencing (SIGS), a phenomenon also referred as environmental RNAi, has been explored by direct application of dsRNAs or small interfering RNA (siRNA) onto host plants or post-harvest products, which leads to silencing of the target microbe/pest gene and confers efficient disease control (Lucena-Leandro et al., 2022). Environmental RNAi is powerful, environment-friend, and can be easily adapted to control multiple diseases simultaneously. As a novel, eco-friendly approach for managing plant pests and diseases, SIGS does not alter the host genome, therefore is widely accepted as an alternative to HIGS that needs genetic modification (Nityagovsky et al., 2022; Parise et al., 2024). Rooted in the natural RNAi process, where RNA molecules inhibit gene expression by neutralizing target mRNA, SIGS provides precise, targeted gene regulation (Niehl et al., 2018; Vetukuri et al., 2021). Early studies highlighted its potential to significantly reduce disease severity in plants, particularly through the suppression of fungal pathogen genes (Koch et al., 2016, 2019; Wu et al., 2024). SIGS has since expanded into pest and disease control strategies, offering high specificity, minimal off-target effects, and high environmental safety (Zotti and Smagghe, 2015; Das and Sherif, 2020). Unlike traditional pesticides, which persist in the environment and harm non-target species, SIGS degrades naturally, reducing ecological and health risks (Aktar et al., 2009; Imfeld and Vuilleumier, 2012). The specificity of dsRNA in SIGS targets pathogen genomes without affecting beneficial organisms such as insects, animals, or soil microbiota, making it a sustainable tool for integrated pest management (IPM) (Zotti and Smagghe, 2015; Christiaens et al., 2020). Studies show that dsRNA degrades before crops reach consumers, addressing concerns about pesticide residues in food and complying with stringent food safety standards (Christiaens et al., 2020). Moreover, SIGS supports soil health, as RNA biodegradation prevents disruption of microbial diversity and soil fertility, contrasting with the long-term detrimental effects of chemical pesticides (Imfeld and Vuilleumier, 2012; Dubrovina and Kiselev, 2019). This aligns SIGS with global initiatives like the European Commission’s Go-Green plan to reduce pesticide use by 2030 (Schebesta et al., 2020; Stojanovic, 2021). Research advances in SIGS include optimizing dsRNA delivery, improving stability, and exploring nanomaterials for enhanced uptake, ensuring its viability for large-scale applications. Despite challenges in foliar absorption due to leaf surface properties and environmental factors like UV exposure, innovations such as clay nanosheets and nanovesicles have extended dsRNA protection in plants (Taning et al., 2020; Hoang et al., 2022). As costs for dsRNA production decrease, products like Ledprona^®^ demonstrate promising commercial potential, positioning SIGS as a key component of sustainable agriculture.

## 2 SIGS for crop protection

**Figure 1:**
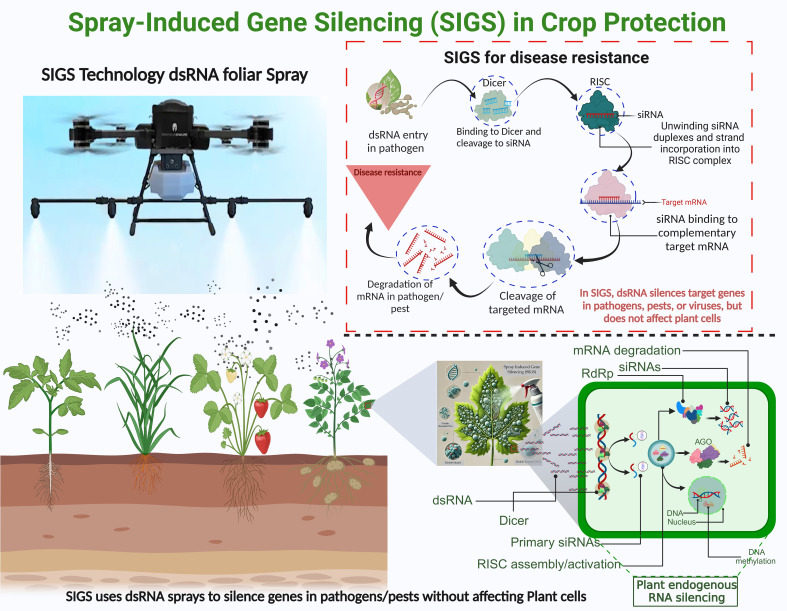
Spray-Induced Gene Silencing (SIGS) technology uses foliar dsRNA sprays to protect crops by silencing target genes. dsRNA is processed into siRNAs by Dicer, incorporated into the RISC complex, and binds to complementary mRNA, leading to its degradation and enhanced disease resistance in plants. The lower panel highlights the molecular mechanism of dsRNA processing and gene silencing within pathogen cells.

The dsRNA-based SIGS technology involves applying dsRNA to plant surfaces, allowing pathogens to absorb it and silence target genes without requiring transgenic plants, making it a faster and more adaptable solution (Beernink et al., 2024). By applying dsRNA to foliage, plants absorb it, triggering RNAi that silences specific genes in pests and pathogens by disrupting their life cycles, boosts disease resistance. Once applied, dsRNA is processed into siRNAs by Dicer, incorporated into the RISC complex, and binds to complementary mRNA, leading to its degradation and enhanced disease resistance in plants (**Figure 1**).

Importantly, SIGS offers a non-transgenic alternative with more immediate applicability, although its effectiveness depends on successful dsRNA uptake (Sang and Kim, 2020). To achieve the desired RNAi response, sprayed dsRNA must ultimately be delivered into responsive cells in the target organism at a sufficient level. The absorption of dsRNA might be relied on endocytosis. On one side, exogenous dsRNA can be directly absorbed by some pathogens or pests. For example, it was demonstrated that *Botrytis cinerea* (*B. cinerea*) can take up external dsRNAs, and applying dsRNAs on the surface of fruits, vegetables and flowers significantly inhibits grey mould disease (Wang et al., 2016). However, the ability to uptake dsRNA varies among different pathogens, e.g., it was observed efficient uptake in fungal pathogens, like *B. cinerea*, *Sclerotinia sclerotiorum*, *Rhizoctonia solani*, *Aspergillus niger* and *Verticillium dahliae*, but no uptake in *Colletotrichum gloeosporioides*, and weak uptake in a beneficial fungus, *Trichoderma virens* (Qiao et al., 2021). Effective uptake of dsRNAs in both *Fusarium oxysporum* and tomato tissues has been presented by fluorescence tracing (Ouyang et al., 2023). In contrast, we found weak uptake of dsRNA in *Fusarium graminearum*, a prevalent pathogen causing fusarium head blight. It was showed that uptake of dsRNA in *Sclerotinia sclerotiorum* through clathrin-mediated endocytosis (Wytinck et al., 2020b). For insects, the dsRNA molecules are absorbed through intestinal uptake following feeding, allowing for systemic spread. This systemic RNAi in nematodes is mediated by multiple SID proteins that are dsRNA specific membrane channels (Whangbo et al., 2017; Wytinck et al., 2020a). On the other side, sprayed dsRNA can be absorbed by plant through different organs and then secreted exosome-like extracellular vesicles to deliver sRNAs into fungal pathogen (Cai et al., 2018b). It was reported that sRNAs can be efficiently taken up and systemically transported to *Malus domestica*, *Vitis vinifera*, and *Nicotiana benthamiana* based on trunk injection and/or petiole absorption, and systemically transportation was strictly restricted to the xylem and apoplast (Dalakouras et al., 2018). Anyhow, for the indirect absorption after plant processing, dsRNA must travel from the surface of a leaf through the waxy cuticle, and then traverse the apoplast, cell wall, and plasma membrane to gain access to the plant cell’s RNAi machinery (Bennett et al., 2020). Once inside the plant cell, the applied dsRNA can move to adjacent cells through plasmodesmata and subsequently to distal cells through the phloem vasculature. Out of cell, trace amounts of dsRNA were detected in plant root excretions and in small brown planthoppers honeydew, and hoppers could transfer dsRNA via vomit (Zhang et al., 2022). Furthermore, ingestion of consumer hoppers could also result in localized RNAi in the midguts of the predator spiders, suggesting transmission along food chain.

Since the discovery of RNAi, advances have enabled its application in crop protection through precise, sequence-specific gene targeting via complementary dsRNA molecules. This specificity surpasses conventional pesticides, allowing for targeted pest management with minimal off-target effects. While synthetic pesticides, first widely used in the 1930s, have improved crop yields and quality, they have also led to growing pesticide resistance, particularly in insects (Mota-Sanchez and Wise, 2024). The advent of RNAi, first elucidated in 1998, marked a significant advancement in reverse genetics and pest management (Fire et al., 1998). As a post-transcriptional gene silencing (PTGS) mechanism, RNAi serves as a natural defense system in eukaryotic cells, targeting and degrading specific mRNA to inhibit gene expression (Fire et al., 1998; Baum and Roberts, 2014). This continued exploration of RNAi-based methods highlights its potential as a sustainable and precise alternative to traditional chemical solutions in modern agriculture. In fact, RNAi technology is increasingly recognized for its potential to manage various agricultural challenges, including insect pests, fungal pathogens, and viruses.

### 2.1 SIGS for insect control

The application of dsRNA for insect control typically involves spraying it onto plant surfaces, allowing it to be directly ingested or absorbed by plants and later ingested by insects during feeding from plants (Pitino et al., 2011). The dsRNA passes through digestive system and enters insect cells, where it is processed by the Dicer complex into small interfering RNAs (siRNAs). These siRNAs are then incorporated into the RNA-induced silencing complex (RISC), which targets and cleaves complementary mRNA sequences, preventing the production of essential proteins for insect survival (Fire et al., 1998; Lundgren and Duan, 2013). This disruption affects vital processes such as growth, reproduction, and metabolism, ultimately leading to insect death (Bachman et al., 2013). As summarized, some studies have demonstrated the effectiveness of dsRNA in managing certain insect pests (**Table 1**). For example, a study by Pinheiro et al. (2020) have reported that after three days of injecting dsRNAs on Sri Lankan weevils for target genes, their transcript levels were significantly reduced (up to 91.4%) whereas, feeding of weevils with targeted dsRNAs showed significant decreases in gene transcript levels and significant mortality of insects treated with Prosα2 and Snf7 dsRNAs (78.6 to 92.7%). In another study, microinjection of dsRNA into the larvae of *Frankliniella occidentalis* thrips effectively silenced key genes (V-ATPase-B, CYP3653A2, and ApoLp-II/I), confirmed at 48 and 72 hours post-injection during the first and second instar stages, improving tissue health and survival whereas silencing CYP3653A2 or ApoLp-II/I increased larval mortality, proving their essential role in vitality (Han and Rotenberg, 2024).However, sensitivity to dsRNA varies across insect orders, with *Coleopterans* (beetles) being highly responsive, requiring only small amounts of dsRNA to achieve significant mortality by silencing key genes (Nitnavare et al., 2021). In contrast, *Hemiptera* (sucking insects) and *Lepidoptera* (moths and butterflies) display more variable responses, depending on their feeding habits and RNAi pathway efficiency (Niehl et al., 2016; Christiaens et al., 2020). For example, foliar application of dsRNA targeting *Leptinotarsa decemlineata* (Colorado potato beetle) genes such as *Actin* and *V-ATPase* resulted in reduced population growth, while plant-mediated RNAi targeting the *CYP6AE14* gene in *Helicoverpa armigera* (cotton bollworm) led to impaired growth and reduced survival (Mao et al., 2007; San Miguel and Scott, 2016). Additionally, dsRNA targeting *V-ATPase* or tubulin in the western corn rootworm agar diet effectively increased *Diabrotica virgifera* larval mortality and/or development stunting (Baum et al., 2007). Similar dsRNA-based approaches have shown promising results in controlling pests like *Myzus persicae* (green peach aphid), *Tuta absoluta* (tomato leaf miner), and *Plutella xylostella* (diamondback moth), reducing their reproduction, survival, and feeding behavior (Pitino et al., 2011; Camargo et al., 2016). Various delivery methods, including foliar sprays, artificial diet feeding, and bacterial expression of dsRNA, have been employed to enhance dsRNA efficacy (Zha et al., 2011; Sang and Kim, 2020; Beernink et al., 2024). Delivering dsRNA via bacteria offers advantages over *in vitro* synthesis, with studies showing significant mortality in *Leptinotarsa decemlineata* larvae fed on *E. coli* expressing dsRNA targeting multiple genes (Zhu et al., 2011). Similar RNAi effects have been observed in pests like *Spodoptera exigua*, *Tuta absoluta*, and *Chilo infuscatellus* following ingestion of bacteria-expressed dsRNA (Tian et al., 2009; Vatanparast and Kim, 2017; Bento et al., 2020). Anyhow, achieving effective gene silencing under field conditions often requires larger quantities of dsRNA.

**Table 1:** Some reports of dsRNA-based SIGS for the control of insects on plants by foliar spray.

Scientific Name	Common Name	Host Crop	Target Gene(s)	Effect and Results	Reference
Acyrthosiphon pisum	Pea Aphid	Pea Plant	Cathepsin L	Reduced reproduction and survival	(Christiaens et al., 2020)
Bemisia tabaci	Silverleaf Whitefly	Hibiscus	TLR7	increased the mortality of whitefly nymphs	(Chen et al., 2015)
Chilo suppressalis	Asiatic rice borer	Rice	ND	Demonstrated 50% mortality	(Jin et al., 2021)
Diabrotica virgifera virgifera	Western corn rootworm	Maize	Snf7	Higher larval mortality, reduced root feeding, protection of maize roots	(Baum et al., 2007)
Heliothis virescens	Tobacco budworm	Tobacco, cotton, tomato	PBAN	Demonstrated 50-60% mortality	(Choi and Vander Meer, 2019)
Hyblaea puera	Teak defoliator	Tectona grandis	HpEcR	Demonstrated 46% mortality	(Kottaipalayam-Somasundaram et al., 2022)
Leptinotarsa decemlineata	Colorado potato beetle	Potato	Actin, V-ATPase	Significant reduction in beetle population, stunted growth, and death	(San Miguel and Scott, 2016)
Manduca sexta	Tobacco Hornworm	Tobacco	DCL1, DCL2, DCL3, DCL4, and DCL6	Increased the gene silencing	(Kumar et al., 2012)
Mythimna separata	Oriental Armyworm	Chinese cabbage	Chi	Increased mortality, reduction in body weight of feeding larvae	(Ganbaatar et al., 2017)
Myzus persicae	Green Peach Aphid	Peach	Actin	Reduced mobility and survival	(Beernink et al., 2024)
Nilaparvata lugens	Brown Planthopper	rice	V-ATPase	Decreased feeding and survival	(Beernink et al., 2024)
Plutella xylostella	Diamondback moth	Cabbage	acetylcholinesterase	Reduced feeding, impaired neurotransmission, significant larval mortality	
Plutella xylostella	Diamondback moth	cabbage, broccoli	AChE	Demonstrated 69-74% mortality	(Sharath Chandra et al., 2019)
Spodoptera frugiperda	Fall Armyworm	maize	Chitinase	Decreased larval survival and growth	(Sang and Kim, 2020)
Tribolium castaneum	Red Flour Beetle	stored grain pest	Chitin Synthase	Impaired cuticle formation and high mortality	(Christiaens et al., 2020)
Tuta absoluta	Tomato leaf miner	Tomato	v-ATPase B, JHBP	Demonstrated 70% mortality	(Ramkumar et al., 2021)

### 2.2 SIGS for the control of fungal plant pathogens

Fungal plant diseases are a major threat to global food security, leading to annual crop yield losses of up to 20% and post-harvest losses of about 10% worldwide (Delgado-Baquerizo et al., 2020). Traditional methods for managing fungal pathogens rely heavily on fungicides, which have contributed to the rise of fungicide-resistant in fungal pathogens (Fisher et al., 2018; Imran et al., 2021; Wang et al., 2023a). To address this, eco-friendly alternatives such as RNAi technologies have gained attention and recent discoveries demonstrated that many fungal pathogens can absorb environmental RNAs, which can then trigger gene silencing of fungal targets with complementary sequences (Koch et al., 2016; Werner et al., 2020; Qiao et al., 2021). This has enabled the development of SIGS where dsRNAs or sRNAs targeting fungal virulence genes are applied to plants to combat infections. Foliar application of dsRNA has been effective in controlling several fungal pathogens by targeting specific genes (Qiao et al., 2021). The uptake of dsRNA by fungi can inhibit growth, reduce pathogenicity by targeting the specific genes and cause mortality (**Table 2**), presenting a promising alternative to conventional pesticides. However, the efficiency of dsRNA uptake varies across fungal species, with some fungi showing negligible uptake, limiting RNAi effectiveness (Zhang et al., 2016). For instance, dsRNA targeting *Botrytis cinerea* genes *DC-L1* and *DC-L2* significantly reduced its growth and virulence in grapes, decreasing lesion size by over 80% (Nerva et al., 2020). In another study, application of sRNA or dsRNA target DCL1 and DCL2 genes in botrytis and demonstrated significant reduction in gray mold disease (Wang et al., 2016). Similarly, targeting ergosterol biosynthesis genes in *Fusarium graminearum* (*F. graminearum*) reduced fungal transcript levels by 58%(CYP51A), 50% (CYP51B), 48% (CYP51C) in leaves sprayed with CYP3-dsRNA and decreased pathogen DNA in barley (Koch et al., 2016). Targeting the ergosterol biosynthesis pathway in *F. graminearum* not only restricted fungal growth but also disrupted respiration, highlighting the link between ergosterol production and fungal metabolism (Koch et al., 2016). Moreover, dsRNA applied to combat *Austropuccinia psidii* (myrtle rust) showed both preventive and curative effects, reducing disease severity (Degnan et al., 2023). Studies also show the potential of dsRNA to target key respiratory genes in fungi, leading to impaired mitochondrial respiration, reduced ATP production, and overall growth inhibition (**Table 2**). For example, targeting mitochondrial *TIM44* genes in *B. cinerea* resulted in decreased ATP synthesis, affecting fungal energy metabolism (Koch et al., 2016). Similar results were seen in *Sclerotinia sclerotium*, where dsRNA reduced respiratory gene expression by 50%, correlating with lower ATP levels and impaired growth (Wytinck et al., 2020b). Fungal pathogens primarily take up dsRNA through clathrin-mediated endocytosis (CME), with studies confirming that inhibiting clathrin-related genes reduces dsRNA uptake and RNAi efficacy (Wytinck et al., 2020b). While CME is the dominant mechanism, other less-understood endocytic pathways may also contribute to dsRNA absorption. Understanding these mechanisms is critical for optimizing RNAi-based strategies to improve fungal disease management in agriculture.

**Table 2:** Some reports of dsRNA-based SIGS for the control of fungal plant pathogens by foliar spray.

Fungal Pathogen	Disease Caused	Host Crop	Target Gene(s)	Effect and Results	Reference
Botrytis cinerea	Gray mold	Tomato, Grapevine	Bc-DCL1 Bc-DCL2	Significant reduction in disease symptoms	(Wang et al., 2016)
Botrytis cinerea	Gray Mold	tomato	Botrytis-specific genes	Reduced gray mold incidence	(Qiao et al., 2023)
Botrytis cinerea	Gray Mold	Grapevine	bcsod1	Decreased disease severity	(Patel et al., 2008)
Botrytis cinerea	Gray mold	‐‐‐	*mgfp4*	Decrease in GFP level > 90%	(Espino et al., 2014)
Colletotrichum gloeosporioides	Anthracnose	Mango, Chili	CgLAC	Decreased fungal virulence and disease incidence	(Zhou et al., 2021)
Fusarium asiaticum	‐‐‐	wheat	Myosin 5	Reduction of pathogen sensitivity to phenamacril with lower infection	(Song et al., 2018)
Fusarium graminearum	FHB	Wheat, Barley	Fg-CYP51	Reduced fungal growth and FHB severity	(Koch et al., 2016)
Fusarium graminearum	FHB	Wheat	Tri5	Reduced fungal infection and improved yield	(Baldwin et al., 2018)
Fusarium graminearum	FHB	Barley	Fg10360, Fg13150, Fg06123	Reduction in disease development	(Kim et al., 2023)
Fusarium asiaticum	‐‐‐	Multiple crops	β1, β2-tubulin,	Effective silencing, enhanced fungal sensitivity	(Gu et al., 2019)
Fusarium oxysporum	Wilting	Tomato, Banana	Fg-ERG11	Reduced fungal growth and wilt symptoms	(Singh et al., 2020)
Fusarium oxysporum	Wilting	Tomato	Tup1	Reduced wilt symptoms	(Fan et al., 2024)
Magnaporthe oryzae	Rice blast	Rice	MoDES1	Inhibition of fungal development and reduced lesion size	(Sarkar and Roy-Barman, 2021)
Puccinia striiformis f. sp. tritici	Stripe rust	Wheat	PsFUZ7	Lower rust infection and fungal development	(Zhu et al., 2017)
Rhizoctonia solani	Sheath blight	Rice	Rstps2	Lower fungal biomass and disease symptoms	(Zhao et al., 2021)
Sclerotinia sclerotiorum	White mold	multiple crops	SsAgo2	Reduced infection and fungal biomass	(Mukherjee et al., 2024)
Verticillium dahliae	Verticillium wilt	Cotton	VdThit	Suppressed fungal growth and disease symptoms	(Wang et al., 2024a)
*Rhizoctonia solan*	tobacco target spot	Tobacco	*endoPGs/RPMK1*	Significant reduction in disease development	(Wang et al., 2024b)

### 2.3 SIGS for the management of plant viruses

Plant viruses are one of the most significant threats and emerging challenge in agriculture, leading to severe crop losses, from stunted growth to total crop failure, impacting both yield and quality (Tatineni and Hein, 2023; Hamim et al., 2023). Conventional control methods often fail due to the high mutation rates of plant viruses, whereas RNAi-based approaches, particularly dsRNA technologies, offer a promising, sustainable alternative for managing viral diseases, and various studies have documented the use of dsRNA for the control of plant viruses (**Table 3**). When plants are infected, viral small RNAs (vsRNAs) are produced by RNAi machinery of plants, which processes viral dsRNAs or hairpin RNAs (hpRNAs). These vsRNAs degrade complementary viral single-stranded RNAs, acting as a natural antiviral defense (Agius et al., 2012). Building on this, dsRNA-based silencing technologies targeting viral genes have been developed, including engineered dsRNAs or hpRNAs (Marjanac et al., 2009; Guo et al., 2016; Hameed et al., 2017). The key advantage of SIGS technology in the control of viral diseases is its ability to provide targeted control of plant viruses with minimal off-target effects on non-target microorganisms (Baulcombe, 2015; Mitter et al., 2017b; Rêgo-MaChado et al., 2023). Once dsRNA corresponding to viral genes is introduced into the plant, the RNAi pathway is activated, leading to viral RNA degradation and suppression of viral replication, without harming beneficial organisms in the ecosystem (Delgado-Martín et al., 2022). This systemic effect can extend protection beyond the treated areas, enhancing overall plant health and resilience (Mitter et al., 2017b). The uptake of dsRNA by plant cells is facilitated through natural pathways like endocytosis or via viral assistance (Mitter et al., 2017a). Inside the plant cells, Dicer-like (DCL) enzymes cleave dsRNA into small interfering RNAs (siRNAs), which are then incorporated into the RNA-Induced Silencing Complex (RISC). This complex cleaves complementary viral RNA, preventing viral replication (Zotti et al., 2018). The RNAi response can spread throughout the plant via the vascular system, amplifying the silencing signal and enhancing viral defense (Singh et al., 2019). The ability of dsRNA to target essential viral genes makes it a versatile tool for managing various viral threats in agriculture (Mitter et al., 2017b; Cagliari et al., 2019). For example, it was demonstrated that SIGS significantly delayed symptoms of Tomato spotted wilt virus (TSWV) (Tabein et al., 2020), and dsRNA stability was enhanced through delivery methods like nanoparticle encapsulation and biopolymer incorporation (Mitter et al., 2017a). Additionally, dsRNA applied to crops like papaya, zucchini, and cucumber significantly reduced viral symptoms and virus accumulation (Delgado-Martín et al., 2022; Rêgo-MaChado et al., 2023). Studies have also shown that dsRNA targeting specific viral genes (e.g., RP gene in pepper against mild mottle virus, HC gene in tobacco against tobacco etch virus, RNA3 gene in alfalfa against alfalfa mosaic virus) can lower viral loads and delay systemic symptoms (Tenllado and Díaz-Ruíz, 2001; Tenllado et al., 2003). Similarly, hpRNA targeting Sugarcane mosaic virus (SCMV) in maize reduced viral load, and dsRNA targeting the Nib gene in potato enhanced resistance to potato virus Y (PVY) (Gan et al., 2010; Sun et al., 2010). Tobacco mosaic virus was also controlled by targeting multiple viral genes (MP, CP, RP, RNA) with dsRNA and hpRNA, significantly reducing viral loads and infection rates (Konakalla et al., 2016). Thus, SIGS-dsRNA technology shows great potential for managing viral pathogens. Advances in dsRNA stabilization techniques, such as UV protection and temperature resilience, will be key to improving the practicality and commercial viability of SIGS in agriculture. Additionally, the immune-priming effects of dsRNA may improve plant resilience to other pathogens and stressors, promoting better nutrient uptake and overall plant vigor.

**Table 3:** Some reports of dsRNA-based SIGS for the control of plant viruses.

Target virus	Host crop	Target Gene(s)	Effect and Results	Reference
Bean Common Mosaic Virus	Tobacco, cowpea	NIb, CP	Reduced infection rate	(Worrall et al., 2019)
Pepper Mottle Virus	Tobacco	RP	Viral load reduction, phenotype resistance	(Tenllado et al., 2003)
Pepper Mottle Virus	Cowpea, tobacco	RP	Reduction of viral load, slight infection	(Mitter et al., 2017a)
Plum pox virus	Tobacco	IR 54	1/10 dilution reduced/no disease symptoms at life cycle completion	(Tenllado et al., 2003)
Sugarcane Mosaic Virus	Maize	CP	Viral load reduction, no or mild systemic symptoms	(Gan et al., 2010)
Sugarcane Mosaic Virus	Maize	CP	Inhibition of SCMV infection	(Gan et al., 2010)
Tobacco Mosaic Virus	Tobacco	RP, MP	suppression of local and systemic viral dissemination	(Namgial et al., 2019)
Tomato Mosaic Virus	Tobacco, quinoa	CP, MP	Lower infection rate	(Rego-MaChado et al., 2020)

## 3 Major advancements in the technology

Recent developments in the use of dsRNAs for plant protection have focused on target selection because optimized targets allow the precise gene silencing of specific pests or diseases, minimizing collateral damage to beneficial microbiota and microorganisms, and ultimately promoting ecosystem health. The integration of biomarkers into the target selection process enables researchers to predict biological responses with greater accuracy, thereby refining SIGS application to enhance the effectiveness while reducing non-target impacts. Simultaneously, advancements in the production methods aim to scale up the availability of these siRNAs, making them more accessible for sustainable agricultural practices. Improved microbial and enzyme-based synthesis techniques, alongside innovations in nanotechnology, significantly enhanced the scalability and practicality of dsRNA applications in agriculture. Furthermore, optimizing dsRNA design, considering factors such as length and nucleotide composition, enhances the silencing efficiency by improving cellular uptake and initiating RNAi more effectively. These advancements not only lower production costs but also improve the stability and delivery efficacy of dsRNA under various environmental conditions, protecting it from rapid degradation. Consequently, these innovations ensure the prolonged effectiveness of SIGS applications, expanding their utility across diverse crops and agricultural settings and opening new horizons in sustainable agricultural production.

### 3.1 Screening and selection of targets

The success of SIGS relies heavily on the precise selection and screening of target genes (Zhao et al., 2024). Identifying the genes that are crucial for survival, virulence, or reproduction of insect pests and plant pathogens is essential for effective control strategies. Advancements in bioinformatics and multi-omics technologies have significantly improved the efficiency of this gene-targeting process (Sigoillot et al., 2012; Ahmed et al., 2015). Among these technologies, high-throughput sequencing and gene expression profiling have enabled the researchers to identify differentially expressed genes during the infection or infestation, making the genes as a prime candidate for silencing. In contrast, comparative genomics have facilitated the identification of conserved genes across multiple pest species, ensuring that SIGS technology maintains a broad spectrum of effectiveness. An illustrative example is the Functional Representation of Gene Signatures (FRoGS) technology, which employs machine learning to enhance target prediction by integrating the gene functions within a comparative analysis framework (Namba et al., 2022) and this approach has demonstrated greater accuracy in identifying the potential therapeutic targets compared to traditional identity-based methods (Chen et al., 2024). Moreover, combining transcriptomic data with genetic perturbation signatures allow the researchers to differentiate between inhibitory and active targets, thereby refining the selection process for SIGS applications in crop protection. Advanced bioinformatics approaches streamline the target identification through in silico analyses, which significantly reduce the time and resources required for experimental validation and this optimization is critical for developing effective and precise SIGS strategies.

### 3.2 Production Methods for SIGS dsRNAs

In SIGS technology, the production methods have evolved to meet the growing demand for effective gene silencing agents in agricultural applications. Traditional methods, such as chemical synthesis and *in vitro* transcription, are now replaced by advanced techniques such as recombinant fermentation and cell-free synthesis, which facilitate the large-scale production of RNA molecules essential for SIGS and ensuring a consistent supply of high-quality silencing agents.

Recombinant fermentation utilizes genetically modified organisms (GMOs) for producing specific RNA sequences, offering several advantages for dsRNA production. Utilizing microorganisms like *Escherichia coli* or yeasts such as *Pichia pastoris* allow the rapid growth and high-density cultures, making this approach more cost-effective and efficient (Vieira Gomes et al., 2018). Additionally, recombinant fermentation provides the precise control over production process, allowing the optimization of conditions like temperature and nutrient supply to enhance the yield and quality (Ross et al., 2024; da Rosa et al., 2024). Thus, recombinant fermentation emerges as a reliable and scalable solution for producing high quality silencing agents in agricultural biotechnology.

Cell-free synthesis (CFS) facilitates the direct assembly of RNA molecules without the need for living cells, presenting lower contamination risks and faster production and this makes the CFS particularly suitable for rapid response scenarios in managing insect pests. Further, CFS allow the quick adjustments of target sequences by simply altering the DNA template which enables the simultaneous production of multiple dsRNA targets within a single reaction vessel (Hough et al., 2022). The scalability of CFS significantly enhances its cost-effectiveness, allowing the rapid production and flexibility in manufacturing processes. Unlike traditional cell-based systems that often require lengthy culture periods and complex scaling procedures, CFS can reduce production time from weeks to hours, facilitating the rapid market response thereby decreasing the labour costs associated with cell maintenance and culture management. Furthermore, CFS systems can be easily scaled up or down without compromising the yield or quality, accommodating the specific production needs without substantial additional costs and this adaptability is beneficial particularly for producing the small batches of specialized products. However, a major bottleneck of CFS is the stability and cost of transcriptase.

Ongoing research on optimizing these production methods to enhance the yield and reduce the costs should be prioritized. Innovations in bioprocessing technologies can improve SIGS production efficiency and promote wider adoption in crop protection strategies against insects, fungi and viruses. These advancements in production methods are fundamental for enhanced efficacy and applicability of SIGS technology in agriculture and ultimately pave the way for sustainable crop protection and promote green agriculture. Once upon a time, cost is one primary factor constraining the large-scale application of SIGS. However, this is not a problem now. In fact, the cost for dsRNA production is quickly decreased along with the industrialization. For example, RNAGri had the ability to produce tons of dsRNA at a cost of 1$/g (Guan et al., 2021), while Greenlight Biosciences further reduced the cost of dsRNA synthesis by combination of microbial fermentation and CFS technologies (https://www.greenlightbiosciences.com/how-do-we-make-rna).

### 3.3 Delivery efficiency of SIGS

**Figure 2:**
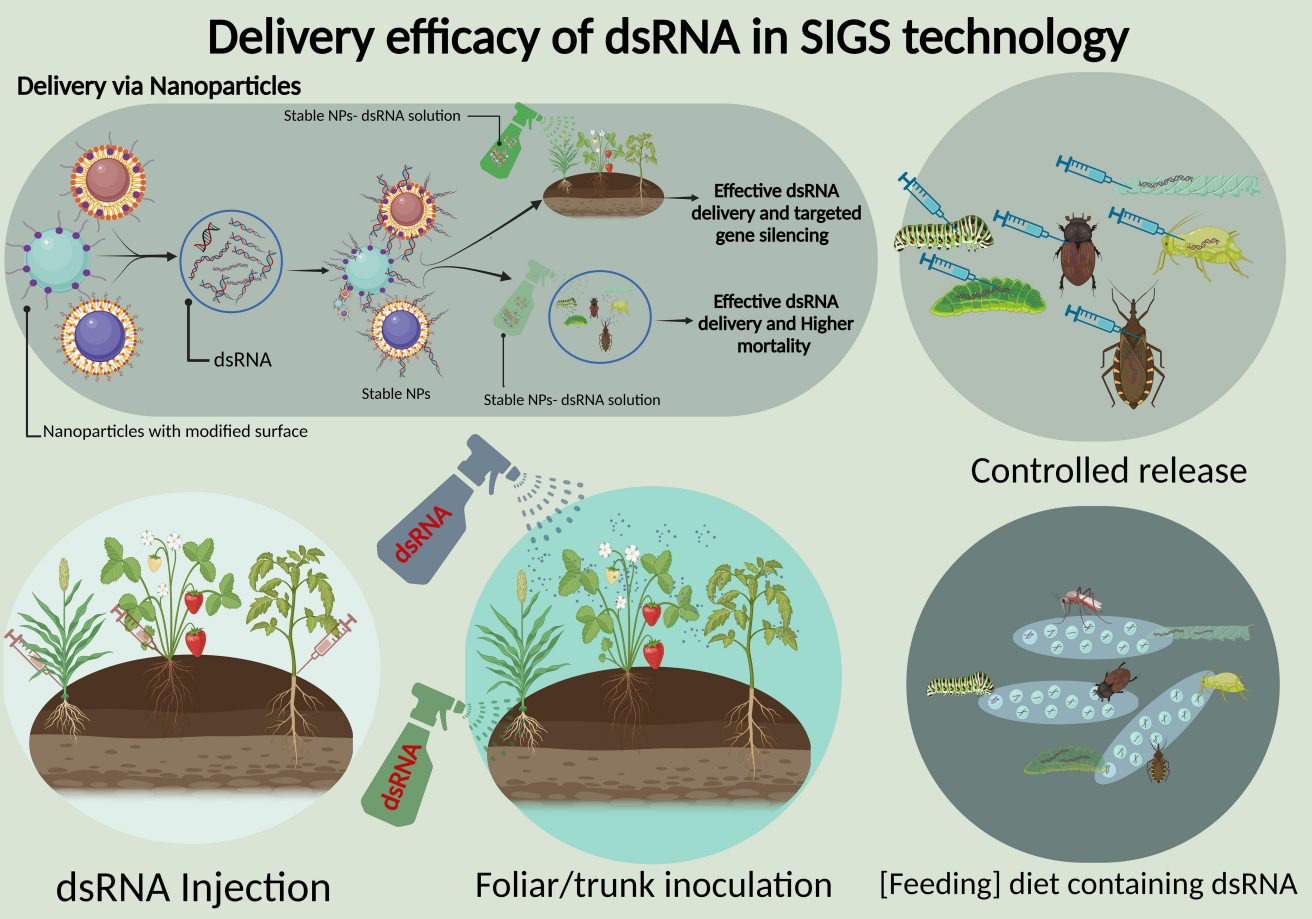
Delivery efficacy of dsRNA in SIGS technology. The figure highlights multiple dsRNA delivery methods: nanoparticle-based delivery for effective gene silencing and insect mortality, controlled release mechanisms, dsRNA injection into plant tissues, foliar/trunk inoculation via sprays, and feeding diets containing dsRNA for targeted pest control. These approaches improve stability, delivery, and efficiency of dsRNA in crop protection.

Efficient delivery of dsRNA remains a core challenge in achieving effective outcomes in SIGS, particularly for protecting the crops from a variety of insect pests and pathogens. The delivery vehicle should safeguard the RNA molecules from environmental degradation, and may also facilitate their uptake into plant tissues because successful delivery requires that dsRNA reaches the target organism in sufficient quantities to elicit a silencing response. As summarized in **Figure 2**, the current delivery approaches include microinjection, controlled release, feeding of dsRNA-containing diet, foliar spray, and trunk inoculation (Wuriyanghan et al., 2011; Pinheiro et al., 2020). The choice of carriers significantly influences the delivery efficiency. Many recent studies have focused on enhancing RNA stability and controlled release through diverse nanocarrier systems and encapsulation technologies (Pal et al., 2024). Thus, various carriers including nanoparticles (NPs), have been explored to improve the stability and uptake by target organisms (Koch et al., 2019).

To optimize these conditions, various NPs, such as chitosan, polyethyleneimine, and layered double hydroxide (LDH) clay nanosheets, have been explored. They can not only protect the RNA from environmental degradation but also demonstrate facilitated absorption by target pathogens thereby significantly increased the gene silencing efficacy. Studies have demonstrated that chitosan and LDH considerably improved the dsRNA stability, and prolonged the protective effects against pathogens for up to 20 days, making them highly suitable for agricultural applications where durability is the most crucial factor (Koch et al., 2019). A study has highlighted the effectiveness of NPs in delivering dsRNA for targeted protection against *Rhizoctonia solani*, the pathogen responsible for rice sheath blight, where *RsAGO1* and *RsAGO2* were identified as the effective targets for dsRNA interference (Wang et al., 2023b). NPs of protamine, carbon quantum dots and graphene quantum dots demonstrated the ability to form stable nanoparticle-dsRNA structures and effectively silenced MGV1 and RAS1 genes of *F. graminearum* (Gyawali et al., 2024). Notably, carbon quantum dots and chitosan/SPc complexes enhanced the dsRNA loading capacity by maintaining the functionality with only a 7% reduction in fluorescence intensity post-nuclease treatment (Wang et al., 2023b). Some other carriers, like liposomes and carbon nanotubes, have also been utilized as NPs offer superior stability and delivery efficiency, which makes them the optimal for SIGS applications because NP carriers reduce the frequency of applications and minimize the chemical runoff into ecosystems (Mittal et al., 2020). This targeted delivery effectively silences the specific genes in pests and pathogens without affecting non-target organisms by reducing the reliance on broad-spectrum pesticides. Additionally, these biodegradable carriers, derived from renewable resources, also mitigate the environmental toxicity associated with traditional chemical treatments.

## 4 Remaining questions and concerns in SIGS

Despite the promising potential of SIGS technology for crop protection, several critical challenges must be addressed to optimize its efficacy and safety. One of the primary concerns is the stability of the RNA molecules utilized in gene silencing, as various environmental factors and enzymatic activities in plants can degrade these RNA molecules. Although researchers have documented that the structural modifications to RNA can enhance its stability, these modifications may also influence silencing efficiency (Dubrovina and Kiselev, 2019). Achieving consistent delivery and expression levels in targeted plants remains a significant challenge, as variations in plant responses to SIGS treatments can lead to inconsistent outcomes. This variability highlights the necessity for further optimization of application protocols and formulations. To tackle these issues, researchers have investigated various encapsulation methods, and embedding dsRNA in protective nanocarriers considerably increased the stability and persistence on plant surfaces (Guo et al., 2022; Pal et al., 2024). For example, the delivery of dsRNA to soybean aphid *Aphis glycines* demonstrated rapid penetration into the body wall within 4 hours with the help of nanocarrier. Through the topical application effectively silenced the target gene expression with the knockdown effects ranging from 86.86 to 58.87% and demonstrated higher mortality up to 81.67% (Yan et al., 2020). Among these nanocarriers, clay nanosheets have demonstrated considerable potential by effectively shielding the dsRNA from degradation due to UV exposure and heat while facilitating a gradual release. This sustained release allows for a prolonged presence of RNA on the plant, thereby maintaining the bioactivity of the molecules and dwindling the gap for gene silencing effects (Mitter et al., 2017a).

Another significant challenge associated with SIGS is the risk of off-target effects. While SIGS is designed to specifically target certain genes, the inherent specificity of RNAi relies heavily on the sequence complementarity between the dsRNA/siRNA and the intended target gene. Unintended interactions with non-target genes can lead to adverse effects on plant health and development, as evidenced by studies documenting off-target silencing due to sequence homology (Chen et al., 2021). To mitigate these risks, bioinformatics tools, including homology-based sequence screening and predictive modelling, are increasingly utilized to refine target selection and minimize off-target effects. Nevertheless, extensive *in vivo* testing across diverse plant species remains crucial for further assessing and mitigating these risks, underscoring the need for robust methods to predict and evaluate off-target activity (Dubrovina and Kiselev, 2019; Vetukuri et al., 2021). As dsRNA might persist in soil and enter the food chain, posing potential risks to biodiversity and human health (Papadopoulou et al., 2020). Thus, addressing these challenges requires developing the biosafety frameworks and advanced predictive models to assess dsRNA persistence and its pathways in non-target organisms (San Miguel and Scott, 2016). The integration of SIGS with traditional chemical controls and advanced nanotechnology offers promising potential for sustainable IPM strategies. This combination may significantly enhance the effectiveness of agricultural practices in managing plant pathogens and pests.

In addition, weed species significantly reduce agricultural productivity by competing for essential resources, such as water, light, and nutrients (Horvath et al., 2023). Their rapid reproduction is aided by traits like deep root systems and allelopathic substance release, which inhibit crop growth and promote pathogens, ultimately increasing cultivation costs (Trognitz et al., 2016). Studies across Europe have identified numerous weed species in various crops, with notable examples including *Fallopia convolvulus* and *Amaranthus retroflexus* (Gerhards et al., 2017; Hofmeijer et al., 2021). A patent has explored SIGS to combat herbicide resistance, such as restoring glyphosate efficacy in *Amaranthus palmeri* (Sammons et al., 2011). An interesting study designed three types of RNAi-based herbicides that specifically silenced endogenous target genes and controlled the growth of *Mikania micrantha* Kunth (Mai et al., 2021). In particular, the study shown that weed leaves turned yellow and eventually wilted after spray of dsRNA targeting Chlorophyll a/b-binding protein. However, genetic similarity between crops and weeds complicates the design of dsRNAs that risking off-target effects in related crops. Addressing the challenges of delivering stable siRNA formulations and enhancing genomic knowledge of weeds is crucial for making SIGS a viable alternative to conventional herbicides (Zabala-Pardo et al., 2022). Despite advancements in RNAi for insect pests and viruses, its application for weed management remains limited, and to the best of our knowledge and based on available literature.

**Figure 3:**
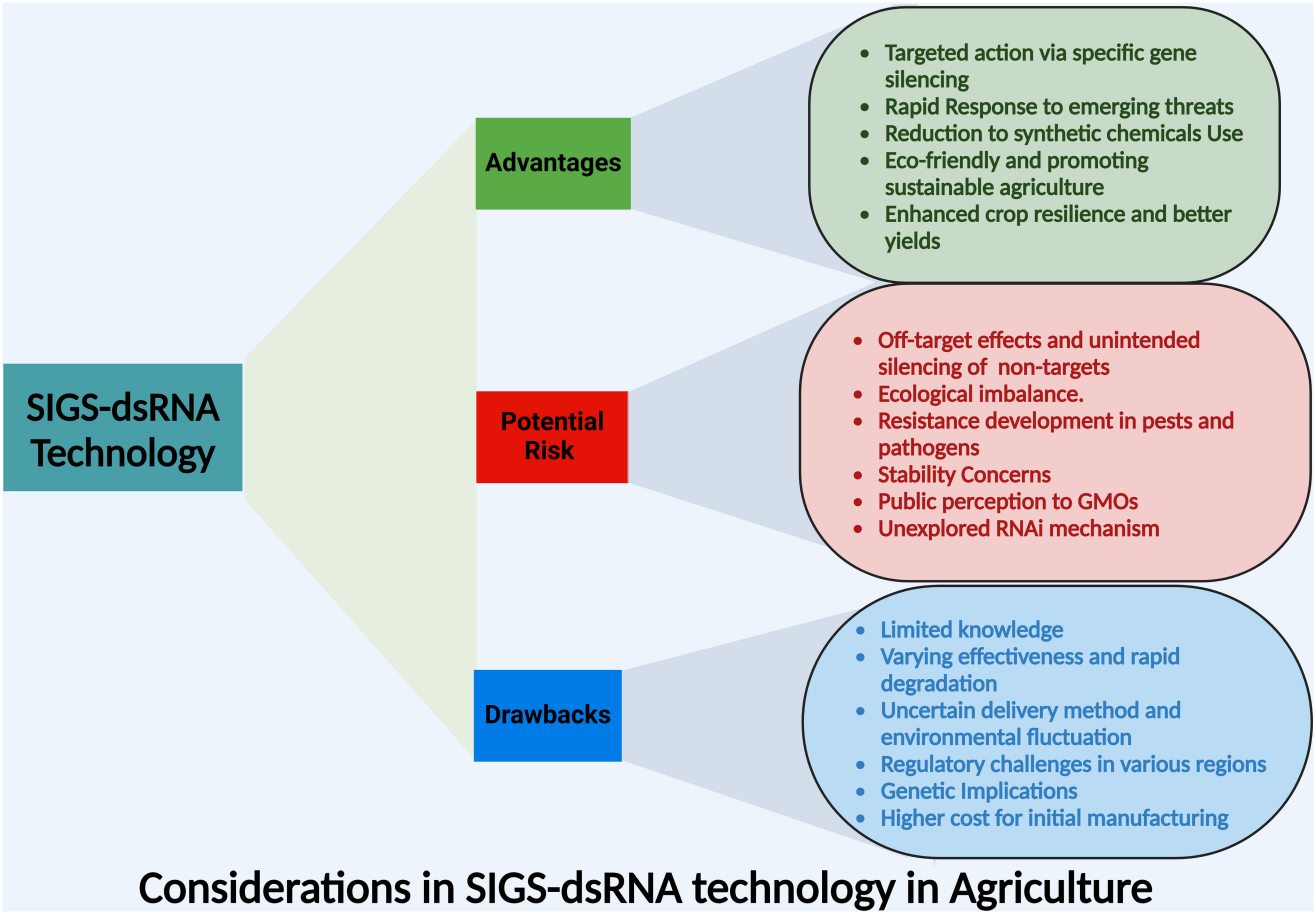
An overview of the core advantages, potential risks and drawbacks of dsRNA application in agriculture associated with SIGS technology for crop protection.

Generally, SIGS technology offers targeted pest control, rapid response, and reduced pesticide reliance, enhancing crop resilience. However, in almost all of these studies pathogens were inoculated simultaneously with or shortly after dsRNA treatment, which is different from the real situation. Under natural conditions, pathogens often have already existed in the plant tissue, therefore limiting the value in application. Meanwhile, most of the studies were conducted using disease models and little field studies were conducted. From a practical point, SIGS also faces challenges like variable efficacy, regulatory hurdles, and high costs, including risks like off-target effects, resistance development, and concerns about dsRNA stability and public perception of genetic manipulation (**Figure 3**). Thus, to enhance the application of SIGS technology, several key scientific questions need exploration, which includes identifying the specific RNA modifications that improve stability while preserving silencing efficiency, refining predictive models for off-target effects to increase accuracy across diverse plant species, and assessing the ecological impacts of dsRNA persistence in soil on non-target organisms. Additionally, understanding how SIGS integration with other pest management strategies can influence the resistance development in target pathogens is vital, and establishing the regulatory frameworks for the safe application of SIGS under field conditions is essential. Thus, addressing these questions will be crucial for optimizing the efficacy and safety of SIGS in sustainable agricultural practices.

## 5 Emerging trends

In green agriculture, fungal and bacterial communities are emerging as effective biocontrol agents in sustainable agriculture due to their eco-friendly performance (Aldayel et al., 2024; Imran et al., 2024). These biocontrol agents produced various volatile metabolites and hydrolytic enzymes that can enhance the efficacy of dsRNA applications for pest and pathogen management because these compounds serve as signalling molecules, triggering plant defence mechanisms and complementing the effects of dsRNA, which targets specific genes in pests and pathogens to inhibit their growth (Imran et al., 2022). Further, these species respond to the volatile compounds emitted from pathogens and upregulate biocontrol-related genes, thereby producing antifungal metabolites (Giorgio et al., 2015; Li et al., 2018). This interaction not only amplifies the inhibitory effects of dsRNA but also bolsters plant resilience against biotic stresses, suggesting that combining volatile compounds with dsRNA strategies could yield synergistic benefits. High-throughput screening methods for identifying effective bacterial biocontrol agents can further enhance the application of dsRNA technologies, allowing simultaneous screening of thousands of candidates and improving overall biocontrol strategies (Hough et al., 2022; Kjeldgaard et al., 2022). Emerging trends in SIGS technology highlight its potential to revolutionize crop protection through innovative applications and methodologies as it enables targeted gene silencing to control pests and pathogens by delivering dsRNA molecules to crops, positioning it as a promising alternative to traditional pesticides. Further, it offers a more sustainable, targeted, and environmentally friendly solution. Current trends in SIGS emphasize overcoming these challenges by integrating new technologies, particularly focusing on nanotechnology and the production of dsRNA by biocontrol agents to enhance the efficacy of RNA-based applications.

### 5.1 Combination with nanotechnology

The integration of nanotechnology into SIGS represents a significant advancement in enhancing the stability, delivery efficiency, and targeted action of dsRNA molecules. Encapsulating dsRNA within NPs protects it from environmental factors that influence its effectiveness under open field conditions, thereby extending its functional longevity. This encapsulation reduces the frequency of applications needed for gene silencing and minimizes the costs. Various NP carriers, including liposomes, biopolymer nanoparticles, and virus-like particles (VLPs), have been explored (Pal et al., 2024) for their potential to deliver dsRNA effectively. For example, liposomes encapsulate dsRNA in lipid bilayers that mimic cellular membranes, facilitating the absorption by plant cells, while biopolymer NPs derived from natural or synthetic polymers offer biocompatibility and controlled release properties, making them more suitable for agricultural applications (Ghosh et al., 2023). Contrary to VLPs, which resemble viruses but lack infectious genetic material, also deliver dsRNA by evading the host defense that typically degrades foreign genetic material and enhances the stability and precision (Sarkar and Roy-Barman, 2021; Ghosh et al., 2023). One notable advantage of NP use is the improved stability of dsRNA in alkaline environments, such as insect guts, where it would otherwise degrade rapidly, as studies have shown that specific NP formulations can protect the dsRNA from high pH and enzymatic changes, prolonging its activity and enhancing its insecticidal efficacy (Qiao et al., 2023). This is particularly relevant for controlling pests with alkaline gut environments, where traditional dsRNA applications may fail without NP protection (Yang et al., 2022). Furthermore, nanotechnology enables controlled release mechanisms that enhance the persistence of RNAi agents on plant surfaces by minimizing their application frequency required for effective pest management (Dhandapani et al., 2022). Thus, the combination of SIGS with nanotechnology not only addresses RNA stability challenges but also paves the way for more targeted and efficient crop protection strategies.

### 5.2 Consideration of mycovirus

As major drivers for controlling host populations and evolution, viruses are potential biological control agents (Wagemans et al., 2022). Mycoviruses are viruses that infect fungi, and some mycoviruses may alter fungal host pathogenicity resulting in hypervirulence or hypovirulence and therefore could be used for plant protection. Hypovirulence-inducing mycoviruses represent a powerful means to defeat fungal epidemics on crop plants. Infections of fungi by mycoviruses are sometimes fatal, as they perturb sporulation, growth, and, if applicable, virulence of the fungal host. A dsRNA chrysovirus-like mycovirus (FgV-ch9) debilitates *Fusarium graminearum*, the causal agent of fusarium head blight (Bormann et al., 2018). The chrysovirus FodV1 also induces hypovirulence of its host *F. oxysporum*, which causes vascular wilt in carnation by decreasing the colonizing efficiency of its fungal host (Torres-Trenas et al., 2019). A novel mycovirus, designated as PtCV1, from the fungus *Pestalotiopsis theae* (*P. theae*), a pathogen of tea, has four dsRNAs as its genome. PtCV1 can significantly reduce the growth rates of its host fungus *in vitro* and abolish its virulence in planta, converting its host fungus to a non-pathogenic endophyte on tea leaves, while PtCV1-free isolates were highly virulent (Zhou et al., 2021). Moreover, the presence of PtCV1 conferred high resistance to the host plants against the virulent *P. theae* strains. In fact, it was found that a large proportion of Fusarium isolates (46%) were infected with mycoviruses and five mycoviruses were shared between *F. graminearum* and *F. culmorum* (Buivydaitė et al., 2024). Therefore, the presence of these hypovirulence-inducing mycoviruses may reduce the fitness of a fungal pathogen and enhance the effectiveness of RNAi-based control. For example, it was found a fungal host’s RNAi machinery is upregulated in the presence of mycovirus that lacks a virus-encoded suppressor (VSR), compared to one that has an active VSR (Zhang et al., 2008).

On the other side, the presence of mycoviruses may disrupt the fungal RNAi and derail the efficacy of SIGS control strategy, which requires functional RNAi in the target cell. This is because some mycoviruses produce VSRs, that disrupt their host’s RNAi (Aulia et al., 2021; Ko et al., 2021). These VSRs either suppress the transcription of key enzymes (like DCL2 and AGL2) or reduce the accumulation of siRNA to repress fungal RNAi (Rodriguez et al., 2022; Myers and James, 2022). For example, *Aspergillus* virus 1816 was capable of suppressing RNAi and this resulted in reduced siRNA in *A. nidulans* (Hammond et al., 2008). Similarly, the *Rosellinia necatrix* mycoreovirus 3 showed VSR activity and suppressed RNAi in *Nicotiana benthamiana*, possibly by less accumulation of siRNA (Yaegashi et al., 2013). Therefore, these VSRs might be novel add-on targets for dsRNA-based SIGS, as silencing them can maintain or even enhance the RNAi machinery of the fungal host. Meanwhile, co-application of mycovirus that lacks VSRs and dsRNA will probably enhance the stability of dsRNA by counteracting degradation, achieving prolonged and effective gene silencing. Moreover, mycovirus can be engineered by inserting the target transcript in both sense and antisense orientations, which can convert pathogenic fungi into hypovirulent strains by silencing the target gene. As reported, a mycovirus, FgGMTV1, has been successfully engineered and efficiently triggered gene silencing in *F. graminearum* (Zhang et al., 2023). This is highly promising, as the strategy combined the infectious property of mycovirus and the RNAi mechanism, also simplified the requirements for production and storage.

## 6 Conclusion and prospects

Plants utilize dsRNA for producing siRNA, driving gene silencing and enhancing resistance to pathogens, and this RNAi mechanism allows crops to downregulate critical genes, effectively preventing their further development while minimizing unintended effects through precise, targeted action. However, enhancing target specificity through multi-omics gene selection approaches can minimize off-target effects by preserving the biodiversity, but for this, long-term field studies are required that will inform the regulatory guidelines. Besides, integrating SIGS with IPM and precision agriculture can reduce crop losses and support soil health and beneficial microbiota, as topical application of dsRNA can trigger a protective response, providing reliable control without the adverse impacts associated with traditional chemicals.

Further research on optimal dsRNA concentration, stability, and delivery systems is crucial for realizing the potential of SIGS in sustainable crop disease management. Efficient carriers, such as nanoparticles and bio-based polymers, can enhance dsRNA uptake, while cost-effective production methods like microbial fermentation and cell-free synthesis make SIGS accessible to resource-limited farmers in the form of powders and gels by simplifying transportation and application. To some extent, recent advancements have addressed the production costs, stability, and off-target effects, positioning RNAi as a promising eco-friendly alternative to synthetic pesticides. In SIGS, pathogen-specific genes are employed to inhibit the growth and pathogenicity, which offers a viable alternative and minimizes the adverse effects to non-target species. However, in-depth addressing of these challenges can effectively scale this technology in a broad spectrum. With ongoing advancements, SIGS is poised to play a pivotal role in meeting global food demands, aligning with the “Third Green Revolution” to ensure effective crop protection and food security.
